# The Role of Healthy Diets in Environmentally Sustainable Food Systems

**DOI:** 10.1177/0379572120953734

**Published:** 2020-12-24

**Authors:** Michael Clark, Jennie Macdiarmid, Andrew D. Jones, Janet Ranganathan, Mario Herrero, Jessica Fanzo

**Affiliations:** 1 Big Data Institute, 6396 University of Oxford, Oxford, UK; 2 1019 University of Aberdeen, Aberdeen, UK; 3 1259 University of Michigan, Ann Arbor, MI, USA; 4 8309 World Resources Institute, Washington DC, USA; 5 2221 Commonwealth Scientific Industrial Research Organisation, Brisbane, Queensland, Australia; 6 1466 Johns Hopkins University, Baltimore, MD, USA

**Keywords:** diets, environmental sustainability, health, food system transitions, solutions

## Abstract

**Background:**

The global food system is directly linked to international health and sustainability targets, such as the United Nation’s Sustainable Development Goals, Paris Agreement climate change targets, and the Aichi Biodiversity Targets. These targets are already threatened by current dietary patterns and will be further threatened by 2050 because of a growing population and transitions toward diets with more calories, animal-source foods, and ultra-processed foods. While dietary changes to healthier and predominantly plant-based diets will be integral to meeting environmental targets, economic, social, and cultural barriers make such dietary transitions difficult.

**Objective:**

To discuss the role of healthy diets in sustainable food systems and to highlight potential difficulties and solutions of transitioning toward healthier dietary patterns. To do so, we synthesize global knowledge and conduct a series of case studies on 4 countries that differ in their social, economic, political, and dietary contexts: Brazil, Vietnam, Kenya, and Sweden.

**Conclusions:**

No single “silver bullet” policy solution exists to shift food choices toward sustainable healthy diets. Instead, simultaneous action by the public sector, private sector, and governments will be needed.

## Introduction

Food systems—The entire range of actors and their interlinked value-adding activities involved in the production, aggregation, processing, distribution, consumption, and disposal of food products—range from local to global in scale. In 2010, the FAO defined sustainable diets as those with “low environmental impacts which contribute…to healthy life for present and future generations…[and] are protective and respectful of biodiversity and ecosystems, culturally acceptable, accessible, economically fair and affordable; nutritionally adequate, safe and healthy.”^1^ Sustainable food systems vary by scale and by local environmental, economic, cultural, political, and institutional contexts. The context-dependent nature of sustainability means that developing food systems that are sustainable at local to global scales will be a complex challenge.

Many international health and sustainability goals—such as the Sustainable Development Goals (SDGs),^2^ the Paris Agreement,^3^ or Aichi Biodiversity Targets^4^—are linked to food systems. In many cases, meeting these goals will not be possible with current food systems. For health, while the global food system provides food for over 7.5 billion people, poor dietary quality is simultaneously the largest source of poor health.^5^ Less than half of the world’s adult population has a healthy body mass (body mass index ≥ 18.5 and < 25) and micronutrient deficiencies are common in low-, middle-, and high-income countries, thereby risking achievement of SDG2 and SDG3.^6^ Air pollution from food production threatens SDGs 3, 7, and 11. For environment, the global food system occupies ∼30% of Earth’s land surface and is the leading source of land use change. Food systems emit ∼20% to 35% of all greenhouse gas (GHG) emissions, threatening global temperature targets (SDGs 13 and Paris Agreement).^7^ Unsustainable rates of fertilizer application and resultant nutrient pollution,^8^ combined with unsustainable water withdrawals,^8^ land use change,^8^ and the overuse and misuse of pesticides^9^ threaten biodiversity targets (SDGs 14 and 15, Aichi Biodiversity Targets) as well as our ability to provide adequate amounts of safe drinking water (SDG 6).

In this paper, we focus on how dietary transitions toward sustainable healthy diets can help achieve health- and environmental-oriented sustainability targets. We first discuss how current dietary trajectories will increasingly impact human and environmental health. We then highlight how transitions to healthier diets could help meet environmental targets. Third, because transitions to sustainable food systems will differ due to local and national contexts, we use a series of 4 case studies to discuss how adoption of healthy diets might contribute to sustainable food system in countries that vary in their environmental, human health, and sociopolitical contexts. Fourth, we discuss how governments and business can shift individuals toward sustainable healthy diets. We conclude by discussing complexities and potential difficulties of creating sustainable food systems as well as some unanswered questions that might hinder or facilitate their development.

## Using Recent Dietary Trends to Gain Insight into Future Dietary Transitions

Diets have changed rapidly over the past several decades.^10^ Here, we discuss dietary trends from 1975 to 2013 using food supply data, which is measured as the amount of food available for human consumption, due to its geographic and temporal coverage. However, it is important to note that consumption is less than food supply and that an increase in food supply may not translate to an equivalent increase in food consumption.

From 1975 to 2013, global average per capita calorie supply increased by 25%.^10^ Supply of certain foods increased more rapidly. For example, per capita supply of animal-source foods (meat, dairy, and eggs), fruits and vegetables, and vegetable oils increased by 40%, 98%, and 80%, respectively,^10^ although it is important to note that consumption of fruits and vegetables remains below-recommended levels despite recent increases in supply.^11^ Supply of processed foods also increased, depicted by the rapid increase in fats and sugars, while changes in per capita supply of staple foods, such as cereals and starchy roots and tubers, have been smaller.^10^

Dietary transitions have occurred at different rates and different times in different countries (Figure S1).^10^ In low-middle and middle-income countries, such as those in North Africa, East Asia, and South and Southeast Asia, nutrition transitions toward diets with more calories, fresh fruits and vegetables, and animal-source foods occurred as populations became more affluent. From 1975 to 2013, supply of animal-source foods (meat, fish, dairy, and eggs) increased more rapidly than other foods, particularly in East Asia (310% increase), South America (72% increase), and North Africa (100% increase).^10^ Per capita supply of fresh fruits and vegetables also increased in these regions, although current consumption often remains below dietary recommendations.^11^ While Figure S1 and the text discuss the proportional change in the per capita supply of calories, it should be noted that absolute supply varies between countries, with a lower baseline supply in low-income countries.^10^

High-income regions, such as Europe, North America, Australia, and New Zealand, have experienced smaller dietary changes since 1975.^10^ This is primarily because nutrition transitions in high-income countries occurred before 1975, and that supply of calories, fruits and vegetables, and animal-source foods was already high. While the supply of fruit and vegetables has increased since 1975, consumption typically remains below dietary recommendations.^10^ In short, recent and on-going nutrition transitions in low-middle and middle-income nations are causing diets to become more similar to those consumed in higher income nations.^12^

The least affluent regions have not yet experienced large nutrition transitions but are beginning to experience them as populations become more affluent and urbanized.^13^ From 1975 to 2013, per capita total caloric supply in sub-Saharan Africa increased 16% from 2130 to 2460 calories per day, while supply of animal-source foods increased 13% from 168 to 190 calories per day.^10^ However, while nutrition transitions have typically been slow in sub-Saharan Africa, some of the more affluent countries experienced more rapid dietary changes: Since 1975, total caloric supply increased by >40% in 9 countries while supply of animal-source foods increased >50% in 13 countries.^10^ Future dietary trends in sub-Saharan Africa are likely to follow similar trends those that have previously recently occurred in more affluent regions.^13^

Diets globally are projected to continue changing in the coming decades, particularly in lower income regions.^14^ Large increases in animal-source foods and for calories from energy-dense (which are often, but not always, nutrient poor foods; eg, oils, animal fats, alcohol, and sugar) are expected. Between 2010 and 2050, average total per capita caloric supply is projected to increase 15%, while supply of meat is projected to increase >25%, supply of dairy and eggs >50%, and supply of calories from oils, alcohol, and sugar >60%.^14^ However, although future nutrition transitions are projected to be largest in many low- and low-middle-income countries, future per capita supply of total calories and animal-source foods in these countries is projected to remain lower than current supply in many middle- and high-income countries.^14,15^

We have necessarily focused on trends in consumption and supply of different food groups due to data availability, although, as discussed later, the health and environmental impacts of foods within these groups can be variable. However, this does not mean that dietary patterns in high-income nations are unlikely to change in the next decades or that trends in food production follow similar trends to food consumption and supply. In contrast, consumption patterns in these countries are changing rapidly but is not captured by the coarse-grained FAO data. For instance, sales of nondairy milks in the United States increased 61% from 2012 to 2017 and are now consumed by nearly a quarter of Brits^16^; consumption of sugar-sweetened beverages is decreasing, often as a result of policy reform^17,18^; and poultry is being substituted for beef and pork in many countries.^10^ In addition, within a country, trends in production may follow different patterns due to increasing international trade. For instance, there is an increasingly large gap between meat production and consumption in some of the world’s highest producing countries such as Brazil, the United States, and Australia.^10^

## Environmental and Health Impacts of Future Diets

### Environmental Impacts

**Figure 1.**
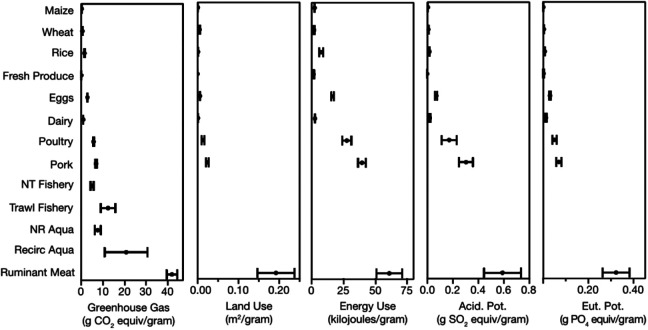
Greenhouse gas, land use, energy use, acidification potential, and eutrophication potential impacts per calorie, g protein, serving, or gram of production of different foods. Dot indicates mean environmental impact, and error bars indicate one standard error around the mean. NR Aqua, non-recirculating aquaculture; NT Fishery, non-trawling fishery; Recirc Aqua, recirculating aquaculture. Figures reprinted from the study by Clark and Tilman.^19^ Impacts follow similar trends when reported per kcal, protein, or serving.

Foods differ greatly in their environmental impacts (Figure 1).^19,20^ Meat from ruminants has the largest environmental impact for most environmental indicators, for instance having GHG emissions, land use, and nutrient pollution impacts 100 times larger than whole grain cereals.^19,20^ Poultry and pork have environmental impacts several times larger than plant-based foods, as do most fish.^19,20^ However, the environmental impacts of fish are variable because of the wide variety of fish and fish production systems.^19^ Dairy and eggs have lower environmental impacts than meat but higher environmental impacts than most plant-based foods.^19,20^

While there can be large variation around the mean environmental impact of a given food, the lowest impact animal-source food typically has higher environmental impacts than the highest impact plant-based food.^20^ While systems that differ in production methodology (eg, intensive and extensive, organic, and nonorganic systems) or location can have different environmental impacts (see Box 1 for further discussion), these differences are small compared to the difference in impacts between animal and plant-based foods.^19-21^ The trend in environmental impact between foods is similar across the different nutritional units (eg, per calorie or per gram) often used to express a food’s environmental impact.^19^

Animal-source foods have larger environmental impacts than plant source foods because livestock are inefficient at converting feed into food.^19,20^ Meat from ruminants has the highest impact for many environmental indicators, in part because producing it requires ∼15 to 20 calories of feed per calorie of edible meat produced,^12^ but also because it is land-intensive and because ruminants release methane,^19,20^ a GHG more potent than carbon dioxide, when digesting food. Pork and poultry production have lower environmental impacts than ruminant meat production, largely because they use less feed and therefore less land to produce the same amount of food, while egg and dairy production typically have environmental lower impacts and use less feed than pork and poultry.^12^

**Figure 2.**
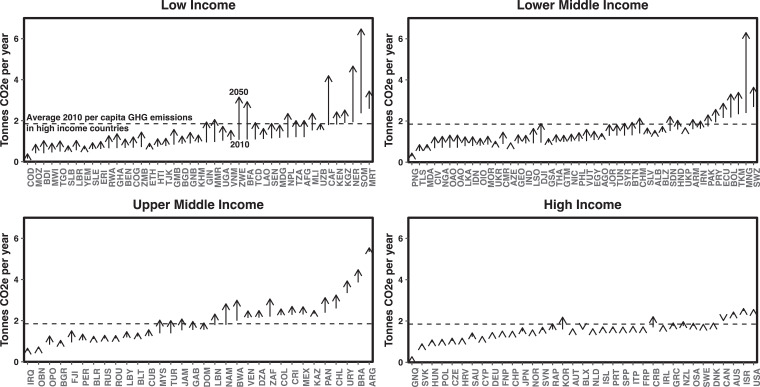
Current and projected per capita diet-related GHG emissions. Countries are labeled by ISO3 code (https://unstats.un.org/unsd/tradekb/knowledgebase/country-code), separated by WHO income groups, and are ordered by current 2010 per capita diet-related GHG emissions within each panel. The start of each segment indicates estimated per capita GHG emissions in 2010, whereas tip of the arrow indicates projected emissions in 2050 if current dietary trajectories continue. Downward facing arrows indicate that per capita diet-related GHG emissions are projected to decrease from 2010 to 2050. Horizontal dashed line indicates average per capita GHG emissions in high-income countries. Data are obtained from the study by Springmann et al.^23^ GHG indicates greenhouse gas.

Dietary transitions to diets that include more calories and larger quantities of animal-source foods are projected to lead to larger environmental impacts (Figure 2).^22,23^ The largest proportional and absolute increases in per capita impacts are projected in currently low-income and lower middle-income countries.^22,23^ Although future diets in low-income and lower middle-income countries are projected to contain fewer calories from animal-source foods than current diets in high-income regions, future per capita diet-related environmental impacts in these regions are projected to be similar to current impacts in upper middle and high-income countries.^24^ This is primarily because food production in less affluent countries has higher environmental impacts per unit of food produced than in more affluent countries,^20,25^ although the gap may narrow as agricultural production practices change. Smaller changes in per capita impacts are projected in upper middle and high-income countries, largely because future dietary changes in these regions are projected to be small.^22^ However, because current per capita diet-related environmental impacts in high-income countries are greater than in most other countries, reducing the per capita impacts in middle- and high-income nations is as important, if not more important (and likely also a more equitable approach), than is slowing increases in per capita impacts in less affluent regions.^22^

Dietary transitions combined with an expected ∼2.5 billion person increase in global population means that global diet-related environmental impacts are likely to increase rapidly. By 2050, global GHG emissions from food production are projected to increase by 50% to 80%, equivalent to 12% to 20% of current global GHG emissions.^23^ Cropland use is projected to increase by 200 million to 700 million hectares, which will also result in GHG emissions from deforestation and other land use change.^23,26,12^ Nitrogen and phosphorus fertilizer applications are projected to increase by 35% and 70% by 2050, respectively,^23^ while pesticide applications are also likely to increase. Increased GHG emissions, agricultural-driven land use change, and nutrient and pesticide runoff will further stress biodiversity, particularly in less affluent regions such, as sub-Saharan Africa and Central and South America, and for species that require large amounts of minimally disturbed habitat,^27^ while increased demand for fish will likely further threaten the already stressed status of freshwater and marine fisheries.^28^

### Health

**Figure 3.**
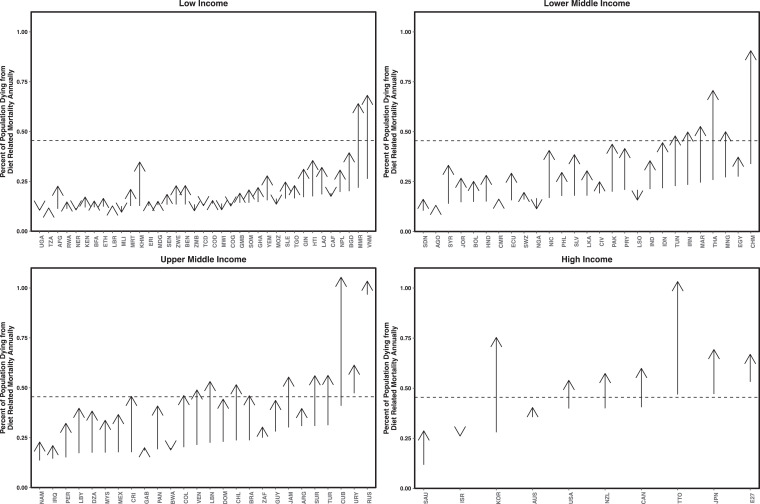
Current and projected prevalence of diet-related related chronic diseases. Countries are labeled by ISO3 code (https://unstats.un.org/unsd/tradekb/knowledgebase/country-code), separated by income WHO income group, and are ordered by current prevalence of diet-related diseases within income groups. Projected increase in percent of the population that has diet-related diseases of diet-related diseases. Downward facing arrows indicate that prevalence of diet-related diseases is projected to decrease from 2010 to 2050. Horizontal dashed lines indicate average diet-related disease prevalence in high-income countries. Data are obtained from the study by Springmann et al.^24^

Future dietary changes are expected to negatively impact human health.^22^ Diet-related disease and mortality risk is projected to increase as a result of increased consumption of foods high in sodium combined with low intakes of whole grains, fruits and vegetables, and weight gain resulting from increased caloric intake above metabolic requirements.^22^ Increased consumption of excess red and processed meat is also expected to increase disease risk but to a smaller extent.^22^ Prevalence of diet-related mortality is projected to increase most rapidly in lower middle and upper middle countries, where diets are changing most rapidly^22^ (Figure 3). In contrast, prevalence of diet-related mortality is projected to decrease in other countries because increased consumption of plant-based food and changes in caloric consumption mean that total caloric consumption is projected to become more similar to metabolic needs.^22,24^

## Consumer Dietary Choice as a Lever to Improve Health and Environmental Outcomes

Consumer-driven transitions toward diets associated with improved health could provide global environmental benefits (eg, ^11,12,22,24^). In this section, we specifically highlight the traditional Mediterranean diet, a lower-meat/flexitarian diet, a pescetarian diet, and a vegetarian diet because these diets have consistently been shown to be associated with reduced disease incidence and risk of mortality in the United States, the European Union, and other high -income nations (see Willett et al^11^ for an in-depth discussion).

These diets are primarily composed of whole grain cereals, fruits, vegetables, nuts and seeds, legumes, healthy oils and fats (eg, oils high in mono- and polyunsaturated fatty acids^29^), ∼1 to 2 servings per day of animal-source foods, and limited amounts of added sugars, sweeteners, and alcohol. The Mediterranean diet is based on what used to be the traditional diets in Mediterranean communities, where prevalence of diet-related diseases was low and life span was among the highest in the world.^30^ Lower meat/flexitarian diets contain small amounts of meat,^11^ pescetarian diets contain fish but no other meat, vegetarian diets do not contain any fish or meat, and vegan diets contain no animal-based foods.

Importantly, there is flexibility for consumers to modify Mediterranean, pescetarian, vegetarian, and vegan diets to accommodate personal food preferences, availability, culture, and socioeconomic values. For instance, the New Nordic Diet is an example of a Mediterranean-like diet modified for the culture, values, and food preferences of the Nordic region.^31^ However, it cannot be assumed that transitioning toward one of these diets will improve health if consumer dietary choices do not also follow healthy eating guidelines or if animal-source foods are replaced with plant-based foods high in refined grains, sugars, and fat.^32^ Likewise, while diets composed primarily of plant-based foods can have low environmental impacts, this cannot be assumed,^33^ as some plant-based foods associated with improved health outcomes can also have a high environmental impact, as is the case with nuts and water use.^34^

**Figure 4.**
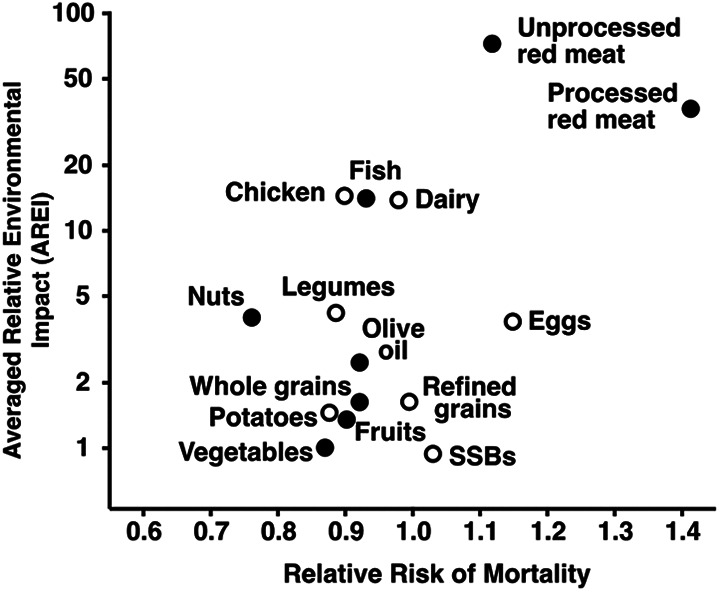
Health and environmental impacts of consuming an additional serving per day of different foods. Health data are primarily drawn from higher income contexts; environmental data are as globally representative as possible given data limitations. Closed circles indicate foods associated with significant changes in health outcomes. Reproduced from the study Clark et al.^34^

Consumers can begin transitioning toward these diets by emphasizing consumption of “win-win” foods—that is, foods that are beneficial for health and have low environmental impacts (Figure 4).^34^ Whole grain cereals, fruits and vegetables, legumes, and most nuts and seeds are good examples. It is also important to identify foods that might be “win-lose” (beneficial for health but have high environmental impacts) or “lose-win” (detrimental to health but have low environmental impacts) to avoid unintended consequences. Fish is a good example of a potential win-lose, being associated with improved health but a relatively high environmental impact depending on how it is produced. Some nuts (especially pistachios and almonds) could be considered win-loses because of their high water use even though they have low impacts for most other environmental outcomes.^35^ Sugar and some oils (particularly those containing trans fats) are lose-wins—bad for health but have low environmental impacts—if they do not result in land use change. If production of sugar and oils results in land use change—as has often occurred in the tropics—then it is more likely that these foods are lose-loses.^20^ Red and processed meat are clear lose-loses, being associated with increased disease risk and having among the highest environmental impacts for all environmental indicators examined. The complexity and tradeoffs of the joint health and environmental impacts of foods is why it is important for dietary recommendations to jointly consider health and environment.

Global adoption of a diet primarily composed of foods that are win-wins—such as a Mediterranean, low-meat/flexitarian, pescetarian, or vegetarian diet—has been estimated to have large benefits relative to projected future diets.^36^ By 2050, the diets highlighted here are estimated to reduce future diet-related GHG emissions by 25% to 60% relative to projected future dietary patterns.^11,23^ The estimated health and environmental benefits of these potential dietary changes vary by region, with larger benefits estimated in higher income countries where current diets often exceed caloric and protein recommendations and contain large quantities animal-source foods.^23,24^ In contrast, smaller benefits are estimated in less affluent regions because diets in these regions contain fewer calories in excess and smaller quantities of foods known to be associated with poor health.^24^ These diets are also projected to offer health benefits, for instance by reducing risk of diet-related diseases such as type 2 diabetes, coronary heart disease, and stroke.^24^ In contrast, increasing consumption of meat, fish, dairy, and eggs could be beneficial in low-income contexts where undernourishment is prevalent: increasing consumption of these foods can be critical in filling nutrient gaps, for example, iron and zinc.^37^ While there is sometimes concern about insufficient protein intake when transitioning to more plant-based diets, this concern is largely unfounded in most countries: Average protein consumption exceeds international recommendations in all but 11 countries where inadequate caloric consumption is common^38^ (see Box 1 for a longer discussion).

## Case Studies

This section presents a series of case studies examining potential pathways and barriers to adoption of sustainable healthy diets in 4 countries. The countries we’ve chosen—Brazil, Kenya, Vietnam, and Sweden—vary in diets, socioeconomic status, political, cultural, economic, and social structures. In each case study, we discuss recent dietary changes, the associated health and environmental impacts, and potential opportunities to transition to healthier and more environmentally sustainable diets. We highlight issues that might be unique to these nations as well as current and potential barriers to adopting healthier and more sustainable diets.

### Brazil—Deforestation to Feed a Growing Desire for Meat

#### Changing dietary patterns in Brazil

Nutrition transitions are occurring in Brazil.^39^ Notably, the increase in consumption of animal-based products, especially meat; the per capita supply of meat has steadily increased from approximately 38 kg/yr in 1985 to 98 kg/yr in 2013.^10^ Traditional diets comprising unprocessed plant-based foods (eg, beans, rice, potatoes, fruits, vegetables) are changing, with people preferring to eat diets containing more animal products and energy-dense ultra-processed foods, which are high in added sugar, salt, or fat.^40^ These dietary patterns are more common in urban areas than in rural areas, where incomes are higher and these foods are more readily available. A survey conducted in São Paulo (2008/9) reported that while approximately 80% of people ate less than recommended intakes of milk and dairy, fruit, cereals, and roots, only 8% ate less than the maximum recommendation for meat and eggs,^41^ with beef being 1 of the 5 most commonly consumed foods.^42^

These dietary patterns are leading to high prevalence of obesity and associated diet-related non-communicable diseases, such as type 2 diabetes, cancer, and heart disease. In Brazil, the prevalence of overweight and obesity increased from 34.1% in 2006 to 48.1% in 2011,^43^ with overweight and obesity being 3 times more common than underweight.^44^ Without any intervention, the prevalence is projected to continue increasing, and that, by 2050, annual obesity-related health care costs will double to approximately $10 billion.^45^ These health outcomes combined with greater recognition of the environmental impact of these emerging dietary patterns, especially the increase in consumption of animal-based products lead to the revision of national dietary guidelines.

The Brazilian dietary guidelines were revised in 2015, which in line with the recent FAO/WHO guiding principles for sustainable health diets,^46^ recommend reducing meat consumption and increasing plant-based foods to improve health and the environment.^47^ A recent study investigating awareness of the link between meat consumption and climate change found that there was greater awareness in Brazil than in the United States and United Kingdom.^48^ People were reportedly concerned about the environmental impact of livestock production, specifically related to deforestation and air pollution. However, they were also concerned that reducing meat consumption would be regressive, which could exclude them from social activities and marginalizing them from peer groups. As with many countries going through economic growth, meat consumption is symbolic of social and economic progress, which can contribute to the reluctance of some people to eat less meat.^49^ This creates multiple health, environmental, social, and economic trade-offs to be considered, not least because livestock production is a major source of income in Brazil.

#### Deforestation to feed a growing desire for meat

Brazil is notable for production of livestock and animal feed to meet the ever-increasing national and global demand for meat. Brazil is the largest exporter of beef in the world. Approximately 20% of global beef exports currently come from Brazil, which is predicted to increase to 23% (2.9 million metric tons) by 2028.^50,51^ The production and export of poultry meat has rapidly increased since the 1980s, with production of poultry now exceeding production of beef. The massive export market was driven in part by the establishment of the Southern Common Market (MERCOSUR) in 1991 that opened export markets, resulting beef exports in Brazil increasing approximately 6-fold and poultry exports almost 12-fold.^10^ Approximately 30% of Brazil’s export revenue comes from agricultural products.^52^ However, the increase in agricultural production and easier access to export markets has come at the expense of the environment, as seen with land expansion and deforestation in the Amazon rainforest, Cerrado, Pantanal, and Atlantic Forest for both grazing cattle and production of feed. In addition to cattle farming, competition for land (and therefore deforestation) is increasing with the growth in other major agricultural exports such as soya (including for animal feed), sugarcane (mainly for ethanol), coffee, and maize.^52^ There has also been a shift from small-scale farming to more intensive and mechanized production systems that has increased use and runoff of agrichemicals such as fertilizer and pesticides.^53^

Agricultural expansion has led to a complex economic and environmental policy conflict over production of commodities. As part of the Paris Agreement in 2015, Brazil made a commitment to reduce GHG emissions by 37% and 43% below 2005 levels by 2025 and 2030,^3^ respectively. Their mitigation strategies included reducing GHG emissions in the agriculture sector (the second largest contributor to GHG emissions in Brazil) and a target of zero illegal deforestation in the Brazilian Amazon by 2030.^54^ At the same time, the More Ranching Plan (MRP 2014) was drawn up, which set out to intensify beef production (based on the concept of land sparing), thereby increasing livestock production through growing herds and higher livestock densities for national supply and for export markets.^55^ The conflict between economic development through agricultural production and environmental degradation continues to grow. As a means to conserve natural ecosystems, The Forest Code was first passed in 1965 requiring landowners to maintain 30% to 80% of their land as native vegetation, then revised in 2012, then over turned by new administration in 2018.

Another dietary recommendation is to avoid eating ultra-processed and mainly eat natural and unprocessed foods. Ultra-processed foods are typically energy-dense, comprising unhealthy ingredients such as sugar, saturated fat, or salt (and are often referred to as “fast foods” or convenience foods).^40^ Production of many of the ingredients in is also having an impact on the environment, including contributing to deforestation for agricultural land, increased monocropping (eg, for soy, oils, and sugar), fertilizer application, and pollution. Global demand for ultra-processed and energy-dense foods is increasingly creating an export market for these ingredients with significant economic for Brazil. As with meat production, there is a complex trade-off between protecting the environment and economic growth.

#### Summary

The current Brazilian food system is currently environmentally unsustainable. Ongoing nutrition transitions towards diets containing more meat and processed energy-dense foods are contributing to an increase in ill-health (eg, obesity and diabetes) and environmental degradation, particularly deforestation and resultant biodiversity loss. The conflict between economic growth from agriculture, cultural values, nutrition security, and the environment risks progress toward improving dietary quality and reducing diet-related environmental impacts. Not only in Brazil but globally, the demand for meat needs to reduce to prevent deforestation. As with other countries, agricultural, nutrition and health, and environmental policies will have to be aligned to create a healthy and sustainable food system.

### Vietnam—The Current and Future Role of Aquaculture

#### The nutrition transition in Vietnam

Like most low- and middle-income countries, Vietnam has undergone a marked nutrition transition in recent decades.^39^ Total daily per capita caloric supply increased 47% from 1975 to 2013 (from 1868 to 2745 calories per day), while per capita consumption of processed food increased nearly 300% from 1999 to 2012 from 10.7 kg per capita in 1999 to 38.7 kg in 2012.^10,56^ Per capita supply of sugar and other sweeteners increased 36% from 1975 to 2013, while consumption of saturated fats, largely from animal-source foods nearly tripled. The rising dominance of animal-source foods in Vietnamese diets is striking. In 1975, per capita caloric supply from animal-source foods was 5.5% (103 calories per day) and had increased to 20.9% (490 calories per day) by 2013. Increasing availability of pork, beginning in the early 1990s, is responsible for most of this increase, with per capita pork supply increasing >600%. Increased supply of other animal-source foods was observed over the same period, with caloric supply of poultry, bovine meat, and fish increasing by 560%, 367%, and 173%, respectively.

These dietary changes have been driven in part by increasing urbanization and per capita affluence as well as changes in retail food environments. Following the “Doi Moi” structural reforms, a series of reforms in the mid-1980s aimed at transitioning Vietnam to a more market-oriented economy, GDP growth averaged 6.4% from 1985 to 2017, while the country’s poverty headcount ratio (percent of the population with <$1.90 GDP Purchasing Power Parity) declined considerably from 52.9% in 1992 to 2.0% in 2016.^57^ These changes in welfare were paralleled by increases in internal migration from rural to urban areas (Vietnam has among the highest rates of rural-urban migration in Asia), cross-border migration from other countries in Southeast Asia, and changes in the retail food environment. The diffusion of modern food retail, characterized by increased supply of energy-dense and ultra-processed foods, reached Vietnam in the late 1990s and early 2000s. These changes were spurred by the liberalization of retail foreign direct investment and food safety concerns, resulting in sales from modern food retail chains increasing by 1900% from 2001 to 2009.^58,59^ The convergence of increased per capita affluence, internal and cross-border migration, and introduction of food retail chains has contributed to recent and ongoing changes in dietary patterns.

Recent changes in dietary patterns have been linked to changing nutritional status. Over the past 2 decades, the prevalence of stunting (defined as low height-for-age) among children under 5 years of age decreased from 61.4% in 1993 to 24.6% in 2015.^57^ This decline has been spurred not only by rapid economic growth but also by specific government efforts to prioritize nutrition, including a National Nutrition Strategy, explicit policies to improve infant and young child feeding practices, and measures to reduce prevalence of micronutrient deficiencies.^60^ Parallel to these reductions in undernutrition, however, is increasing prevalence of overweight and obesity. Between 1980 and 2013, prevalence of overweight and obesity doubled from 6.2% to 12.3% among adult women (≥20 years), tripled among adult men from 4.3% to 13.6%, and increased by 500% since 2000 among preschool-aged children to 4.8% in 2010, which is similar to prevalence of childhood overweight in other countries in Southeast Asia.^57,61^ While prevalence of overweight and obesity among adults is less than half that of most neighboring countries, the marked increase observed in recent years is of concern. Like many lower middle-income countries, Vietnam currently faces multiple burdens of malnutrition. Recent and unprecedented changes in Vietnam’s food system offer both challenges and opportunities to meeting global sustainability targets, and fisheries in Vietnam are uniquely positioned to guide progress toward these targets.

#### Fisheries and aquaculture in Vietnam

Nearly 8 million people, or about 9% of Vietnam’s population, derived their main income from the fisheries sector in 2012.^62^ The economic importance of Vietnam’s fisheries is perhaps not surprising given the country’s more than 3000 kilometers of coastline, numerous inland capture fisheries, and a rich food culture in which fish and fish sauces play a central role. Since the Moi-Doi reforms, fish production in Vietnam increased more than 500%.^10^ By 2020, fish production is expected to reach 7 million tons, with much of this production destined for export, because of a recent 5-year government plan aimed at increasing investment in and supporting development of major fishing centers.^62^ Fish exports have increased rapidly since the Moi-Doi reforms, increasing from 4% of production in 1985 to 47% in 2013,^63^ and currently accounting for an estimated 4% to 5% of Vietnam’s GDP.^64^ While recent increases in fish production have been economically beneficial, they have not occurred without negative environmental impacts. Vietnam’s marine and inshore fisheries, on which millions of low-income fishers depend for their livelihoods, are consistently over exploited.^65^

Aquaculture has become a key strategy for increasing fish production in Vietnam. In 1985, aquaculture accounted for 16% of overall fish production but accounted for 60.4% in 2013 (130 000 tons in 1985 to 3 220 000 tons in 2013).^10,63^ By 2023, domestic aquaculture production is expected to reach 5 million tons.^66^ Brackish water production of whiteleg shrimp (*Penaeus vannamei*) is widespread, as is freshwater, semi-intensive monoculture production of catfish (*Pangasius* subspecies) and tilapia.^63,65^ Integrated polyculture systems in ponds or rice fields producing carp are common, as is marine aquaculture production of finfish and mollusks to a lesser extent.

Reliance on aquaculture production to meet increasing demand is not a panacea and has raised numerous environmental concerns. While freshwater aquaculture in the form of integrated polyculture systems is largely environmentally sound and contributes to farm diversification, coastal aquaculture has contributed to loss of mangroves through wetland conversion (which also emits GHG emissions and reduces protection against coastal flooding), water quality deterioration through discharge of effluent, adverse impacts on wild fisheries due to collection of wild seed, and dramatic increases in the use of “trash fish” (low-value fish that are not often used for human consumption) as feed in aquaculture systems.^65^

#### Aspirations for sustainable diets and the role of fisheries and aquaculture

As Vietnam continues to experience economic growth and macroeconomic stability,^67^ demand for animal-source foods will continue to rise. Solutions that meet increasing demand while preserving and protecting natural ecosystems and supporting the livelihoods of farmers and fishers are required. Proper management of fisheries and aquaculture is essential, and several actions might be considered: (1) protection of and investment in inland fisheries for poor and landless rural dwellers; (2) improved aquaculture management practices, especially in the shrimp production subsector, including improved disease surveillance systems, improved environmental performance, technical support for production of improved quality feed and seed, and decreased mangrove removal; and (3) improved management of overexploited offshore fisheries in the country’s exclusive economic zone, including demarcation of fisheries, monitoring of marine resources, enforcing license limitations, defining gear restrictions, allocating rights-based access to fisheries, and supporting democratic fisheries associations within the country (ie, the Fisheries Association of Vietnam [VINAFIS]).^65^

Implementing measures to increase the environmental sustainability of fish production will be important. Freshwater use in aquaculture production remains a significant challenge, and improvements in land, feed, and energy use as well as reductions in diseases, water pollution, and gene contamination from aquaculture to wild populations need to be addressed to lower aquaculture’s environmental impacts.^68^ In Vietnam, the changes in production management practices noted above are priorities, as is increasing access of aquaculture production to low-income households.

The environmental consequences of projected dietary changes in Vietnam must also be assessed in relation to sustainability targets. Dietary changes are projected to result in an increase in per capita diet-related GHG emissions of 45%, largely due to an increase in consumption of beef, pork, poultry, and other animal-source foods, while land use, water use, and nutrient application and pollution are also projected to increase.^23^ Therefore, demand-side interventions to shift diets away from higher intakes of pork and beef will likely be needed in addition to supply-side management solutions that target production practices. If populations are unwilling to substitute plant-based foods for meats, promoting fish consumption is another potential way to reduce diet-related environmental impacts. Farmed finfish and filter-feeding carp and mollusks, for example, use similar or less feed and can have much lower environmental impacts than other meats,^19^ while filter-feeders have the added benefit of simultaneously improving water quality.

#### Summary

Vietnam is experiencing a double burden of malnutrition where undernutrition and overnutrition are common. Current nutrition transitions toward diets with more calories and animal-source foods, if continued into the future, will likely reduce the prevalence of undernutrition and inadequate caloric intakes but at the cost of increased prevalence of overnutrition and diet-related environmental impacts. Increasing fish consumption in place of increased consumption of higher impact animal-source foods such as pork and ruminant meat could reduce diet-related environmental impacts. However, increases in fish production will likely need to come from aquaculture systems rather than inland or marine fisheries as many fisheries are already overexploited. Increasing fish production could also have economic benefits: fish production is the main source of income for 8% of Vietnam’s population, while improving the sustainability of existing fishery and aquaculture systems would likely improve the likelihood of long-term economic security of individuals who produce fish. In total, Vietnam’s positive economic growth, abundant natural resources, and advances in reducing undernutrition leave it well-positioned to transition toward a sustainable dietary future. Investments in fisheries and the changing role of fish in Vietnamese diets relative to other animal-source foods will likely play a significant role in determining the success of this transition.

### Kenya—Balancing the Role of Livestock

Since 1961, Kenya’s population has increased by nearly 500% (from 8.1 to 48.5 million), putting pressure on the national food system.^10^ Despite a >60% increase in land devoted to crop production, total per capita caloric supply decreased from 2249 to 2160 calories per day.^10^ Per capita meat supply also decreased (from 96 to 85 calories per day) but per capita milk supply increased from 123 to 165 calories per day. Food availability is variable throughout Kenya, with higher availability in urban than rural regions, and for higher income than lower income populations.^69^ While food availability in urban areas is typically higher than in rural areas, food and nutrition security in low-income urban populations is particularly low, an issue that is likely to become increasingly problematic as lower income rural populations migrate to urban centers.^69^

Despite recent changes to the food system and increases in per capita affluence, malnourishment in Kenya remains prevalent. Over 24% of the population is undernourished, with higher proportions among rural and low-income urban populations.^69^ Micronutrient deficiencies are also widespread: Over 75% of the adult population is zinc deficient, 15% is anemic, with higher rates of undernourishment for rural and low-income urban populations, as well as among pregnant women. However, Kenya, as do many other lower income countries, faces a double burden of disease. Overweight and obesity prevalence exceeds 25% and has been increasing by 0.5% per year over the previous decade.^57^ Unequal food distribution and caloric consumption combined with inadequate consumption of fruits, vegetables, nuts and seeds, and whole grain cereals are hurdles to achieving a food system that nurtures human health.^70^

Recent changes to the food system have stressed Kenya’s natural capital. Over 60% of the world’s large mammals (body mass >10 kg) and 25% of the world’s large birds (body mass > 2 kg) are found in Kenya.^71^ While many of these species are not yet threatened with extinction, recent increases in human population sizes, livestock grazing intensities, fence construction to delimit pastures, and hunting and poaching have been linked to population declines for many of these species.^71^ The Coastal Forest is an ecosystem that has been particularly effected by Kenya’s agricultural sector, having lost >90% of its original extent.^72^ Kenya’s fish are faring no better: fish captures, introduction of invasive species, and pollution from surrounding agricultural lands have resulted in rapid changes in fish community composition in Lake Victoria, the world’s second largest freshwater lake.^73^

Agriculture in Kenya is an important source of economic security. Nearly 40% of Kenya’s population is employed in agriculture, and agriculture accounts for over 30% of Kenya’s GDP.^57,74^ However, while agriculture positively benefits nutrition and economic security in regions with affordable inputs and access to markets and innovation,^75^ agriculture is simultaneously a major driver of environmental degradation in Kenya, including habitat loss, biodiversity declines, GHG emissions, and nutrient pollution resulting from fertilizer application. A food system transformation that addresses the current and ongoing health, economic, and environmental aspects of Kenya’s agricultural sector will require multisectoral collaboration so that health-, environmental-, and economic-oriented policies are not antagonistic.

#### Kenya’s current agricultural system

Changing Kenya’s agricultural and food systems will be integral in improving diet-related health and environmental outcomes while maintaining food culture and economic security. Most food production in Kenya comes from mixed crop-livestock systems^76^ consisting of smallholder units with 1 to 4 cows and with several plots of crops grown in intercropping. Maize, beans, and some vegetables are typically grown, both for home consumption and for selling. Milk is the predominant animal-source food—accounting for ∼50% of protein supply from animal-source foods—and is primarily produced for home consumption.

Because many families farm for subsistence and because 80% of food is sold through informal markets (eg, food distribution is often limited subnationally),^77^ improving productivity on smallholder and subsistence farms will be integral. However, smallholder farming is difficult and problematic but is also often the only option for rural populations in Kenya and elsewhere sub-Saharan Africa. Among the difficulties is the need to strike a balance between competing objectives: maximize labour productivity, provide livelihoods, and reduce land and soil degradation to ensure productivity for future generations. Balancing these is becoming more difficult, largely because of declining farm sizes and naturally low levels of soil fertility. Declining farm sizes is a critical restraint to long-term agricultural production because it prevents replenishment of soil fertility through fallow rest periods^78^ and is antagonistic to food and economic security because a smaller amount of land needs to provide for the same amount of people. Poverty characterizes many subsistence households and threatens the hope for a better standard of living for rural populations.

Production of cash crops might be viewed as a potential route to increase household income and thus reduce food insecurity. Elsewhere in sub-Saharan Africa, production of cash crops has not had a consistent impact on food security.^79^ In some locations, cash crop production increases food insecurity by competing with nutrient-dense foods land. Conversely, in other locations, cash crop production can decrease food insecurity by increasing household income. While there is limited evidence on the association between cash crop production and food insecurity in Kenya, it is likely that this association in Kenya is similarly complicated as it elsewhere in sub-Saharan Africa.

#### The potential for improved subnational food distribution

Increasing capacity for food distribution could help alleviate food and nutrition insecurity. Because most of Kenya’s food production is commercialized through informal and local markets, food and nutrition security is often tied to the ability of communities to produce food.^77^ While reliance on locally produced foods is not inherently detrimental to nutrition security, high rates of undernutrition in Kenya indicate that many communities cannot currently provide adequate nutrition. Decreasing farm sizes and expected increases in climatic variations (and thus crop production) threaten to further exacerbate this problem.^80,81^ Increasing capacity for food distribution has potential to mitigate the nutrition impact of and increase resilience to future shocks in agricultural production.

#### The potential for agricultural intensification

Agricultural intensification could be beneficial to food and economic security in Kenya and also has the potential to have environmental benefits including but not limited to reduced land use change. Average crop yields in Kenya are much lower than potential yields, or what yields could be under ideal cropping conditions.^82^ Crop yield gaps, or the difference between current yields and potential yields, are particularly large for staple cereal grains, with current maize, millet, and sorghum yields being 38%, 40%, and 25% of potential yields, respectively. For environment, agricultural land use change is one of the major stresses to biodiversity; increasing crop yields could help decrease future rates of land use change.

Altering agricultural management to more efficiently use existing resources (eg, fertilizer, manure, water, etc) could help improve crop yields. Adjusting timing of nutrient applications such that they match crop nutrient demands could increase crop yields while decreasing nutrient runoff from fertilizer application, although improving and increasing access to agricultural inputs will be needed to do so. Fertilizer subsidy programs in other sub-Saharan African countries have resulted in large crop yield increases in other countries, including in Malawi, Rwanda, Zambia, Ghana, Mali, and Senegal.^83^ A similar program, but tailored to Kenya’s food system, is one approach to improving productivity on existing agricultural lands. In regions where fertilizer access is likely to remain limited, increasing crop yields is possible by integrating nitrogen-fixing legumes or nitrogen-fixing trees into existing agricultural landscapes. Field trials in Kenya found that rotating cereals with legumes increased crop yields by 17% to 24% and economic profits by 32% to 49%.^84^ However, incorporating legumes into crop rotations will require consideration of existing dietary preferences, food traditions, and economic production values, with preference being given to legumes—such as Rosecoco or kidney beans—that are a part of existing food culture and have high market prices.

Increasing production on existing agricultural lands is not a panacea. Intensification via increased fertilizer application can increase rates of fertilizer runoff, potentially leading to biodiversity declines and decreased water quality, particularly if fertilizers are applied in excess.^85^ Some of the negative impacts of increased fertilizer application can be mitigated by agricultural outreach programs and adopting management strategies such as cover cropping.^86^ Similarly, increasing food production does not ensure increased food availability for Kenya’s most at-risk populations, especially those that do not produce their own food. Low-income urban populations, for instance, currently experience among the highest rates of food insecurity,^69^ but increasing food production may not alleviate food insecurity for this population.^87^

#### Summary

Kenya faces a double burden of under- and overnutrition, and recent changes to the food system have stressed Kenya’s natural capital. Population growth and increasing affluence will likely further stress the health and environmental sustainability of Kenya’s food system. Increasing crop and livestock yields through improved management and access to agricultural inputs has potential to improve food and economic security while avoiding the negative environmental impacts of land use change and habitat conversion. Finding and implementing policies that positively affect the food, economic, and environmental sustainability of Kenya’s food system will be a difficult challenge.

### Sweden—Reducing Already Large Per Capita Impacts

Sweden, like many other high-income countries, has large per capita diet-related environmental impacts, primarily because of high consumption of meat, dairy, and eggs.^23^ Current dietary patterns in Sweden are also associated with poor health, partially because of excess consumption of calories as well as inadequate consumption of fruits, vegetables, nuts and seeds, legumes, and whole grain cereals.^70^ Shifting dietary patterns in Sweden and other high-income countries will be integral to meeting sustainability targets.^11^

#### The new Nordic diet

Sweden’s government integrated environmental sustainability and helped develop the “New Nordic Diet,” at least partially in response to high diet-related environmental impacts.^31^ The New Nordic Diet is designed to be healthier (and has been shown to be associated with reduced risk of diet-related diseases^88^) and have lower environmental impacts than current dietary patterns while being culturally, gastronomically, and regionally appropriate. One aspect of the New Nordic Diet is to source locally produced foods for food culture and sovereignty, although locally sourced foods are not necessarily more environmentally sustainable than those produced further away (Box 1). The New Nordic Diet is characterized by high consumption of fruits (>300 g/d) and vegetables (>400 g/d), with particular emphasis on locally available produce such as berries, cabbages, root vegetables, and legumes, potatoes (>140 g/d), whole grain cereals (>75 g/d), nuts (>30 g/d), fish and shellfish (>43 g/d), free-range livestock (85-100 g/d), and some wild game.^31^

Adopting the New Nordic Diet will require a significant transition from current diets, including large reductions in meat (∼50% reduction) paired with increases in fruits (∼30% increase), vegetables (∼130% increase), and nuts (150% increase).^31^ Because sourcing foods locally and regionally is an important aspect of the New Nordic Diet, consumption within food groups will need to change to emphasize foods produced regionally, such as berries, apples, plums, pears, root vegetables, and brassicas such as cabbages and brussel sprouts. If the New Nordic Diet were to be widely adopted in Sweden and elsewhere in the Nordic region, regional food production systems would need to change to meet changing consumer demand.

#### Is the new Nordic diet adequately ambitious?

The potential benefits of adopting the New Nordic Diet in Sweden are unlikely to be sufficient to meet environmental sustainability targets. Larger reductions in meat and dairy, as well as changes in agricultural production practices, will be needed.^11^ Reducing consumption of meat, dairy, and calories (often achieved by reducing consumption of sugars, unhealthy fats, and processed foods) to be in line with international recommendations while simultaneously increasing consumption of fruits, vegetables, and other plant-based foods is estimated to have large environmental benefits in Sweden, including reducing per capita diet-related GHG emissions by 70% and cropland use by 28%. These same dietary transitions have also been estimated to improve diet-related health outcomes in Sweden, including a 21% reduction in premature deaths avoided.^24^

#### Leading the way for other high and middle income countries

The Swedish government, and Nordic Region in general, are uniquely positioned to provide insight into how governments in high- and middle-income countries might promote transitions toward healthier and more environmentally sustainable food systems. While adoption of the New Nordic Diet has been associated with reduced environmental impacts and can help improve health outcomes,^89,90^ it remains unclear how widely the New Nordic Diet is adopted because it is not apparent whether Swedish government (and Nordic Region) has not collected these data. Data on adoption of the New Nordic Diet, as well as the effectiveness of policies incentivizing adoption of the New Nordic Diet, will provide insight into which policies promote transitions to healthier and more environmentally sustainable diets might be most effective if implemented in other countries.

In general, diet-related health and environmental impacts in high-income countries are among the highest in the world (Figures 2 and 3).^24^ Small dietary changes in these countries (and also in higher income populations of less affluent countries), such as substituting poultry or pork for an equivalent amount of ruminant meat could have large environmental benefits and moderate health benefits.^20,91^ However, more ambitious dietary transitions, specifically reducing consumption of total calories, and in particular calories from meat and dairy, would likely be needed to meet environmentally focused sustainability targets.^23^

Diets in many middle-income countries, such as in Brazil and Vietnam, are rapidly transitioning to become similar to those in Sweden and other high-income countries. Such transitions are linked with increased prevalence of diet-related diseases and increased diet-related environmental impacts. Avoiding these trends and associated impacts will be necessary. Emphasizing consumption of fruits, vegetables, and other plant-based foods and moderation of meat, dairy, and eggs, for instance through following dietary guidelines and other policies could have large global benefits.

#### Summary

Diet-related health and environmental impacts in Sweden are large. In response, the New Nordic Diet was designed and implemented by the Swedish and other Nordic governments to improve diet-related outcomes. Yet, from an environmental perspective, the New Nordic Diet is not adequately ambitious to meet sustainability targets: larger reductions in meat, dairy, and eggs would be needed, improvements in farming practices, and reductions in food loss and waste will also be needed. While most high-income countries and middle-income countries do not incorporate environmental sustainability into dietary guidelines, diet-related environmental impacts in high-income countries are among the largest in the world, while diet-related impacts in middle-income countries are rapidly increasing. Incorporating environmental sustainability outcomes into national dietary guidelines has the opportunity to shift diets to become healthier and have lower environmental impacts than what they would likely otherwise have.

## How to Drive Shifts to Sustainable Diets?

What we eat has a profound impact on health and environmental sustainability.^11^ Reducing consumption of red meat, especially ruminant meat, in regions where consumption is above nutritional recommendations would likely provide large environmental benefits.^11^ At the same time, increasing consumption of fruits, vegetables, whole grain cereals, and nuts and seeds would improve human health in most world regions.^70^ While changing diets will not be easy, doing so is necessary to meet the targets set in the SDGs. As discussed previously, consumer choice is a key determinant of diet-related health and environmental outcomes. However, consumer awareness of diet-related health and environmental impacts is not always adequate to shift diets toward more positive outcomes if awareness is not supported by additional actions. How then can governments, businesses, and nongovernmental organizations help drive shifts toward sustainable healthy diets?

### Going Beyond Providing Information and Education

Strategies to shift food choices typically rely on distributing information, but there is limited evidence that this has influenced consumers’ choices in the past.^92,93^ Information distribution strategies include front- and back-of-the pack nutrition labels, dietary guidelines, public health campaigns about the benefits of different food types, and calls for abstinence (eg, vegetarianism or Meatless Mondays). Instead, food choices are influenced by multiple interacting factors, including a food’s price and taste, the age, gender, social identity, and cultural values of the food purchaser, food geography, access to supermarkets and restaurants, and exposure to marketing and media.

Considering how people make choices about what to eat, it’s not surprising that information is insufficient to shift dietary choices. Consumers tend to be highly routinized in their purchasing and consumption habits, especially in retail or food service environments.^94^ Few people notice information and even fewer remember and respond to it. What ends up on the plate is more often a result of habit and unconscious mental processing, than rational, informed decisions.

To drive large-scale dietary shifts, governments, business, and civil society need to expand their repertoire of interventions beyond information distribution. This includes investing to understand the motivation behind consumers’ food choices, using approaches, such as behavioral change and marketing strategies, to design interventions that work in tandem, and policy implementation. However, given the limited experience of shifting diets at large scales, it is important to adopt an experimental approach to interventions, developing baselines of current diets, setting targets, and monitoring outcomes.

### What Actions Can National Governments Take?

Much of the early action to shift diets has been in high-income countries. Yet, given the large and growing health and environmental footprint of the food system, all governments need to act, especially if the health and environmental problems experienced by high-income nations are to be avoided. What actions can governments take to shift diets to become healthier and more sustainable?

First, governments can assess the economic case for shifting to more sustainable diets. The health and environmental costs associated with poor dietary quality are large. For example, in China, the indirect costs of overweight and obesity due to disability and mortality were estimated at 3.6% of GNP (Gross National Product) in 2000 and are projected to grow to 8.7% of GNP in 2025 as diets shift to include more calories, animal-source foods, and ultra-processed foods.^95^

Second, governments can set measurable targets for transitioning to healthier and more sustainable diets. Country-specific information on the economic costs of unhealthy and unsustainable diets coupled with the benefits of shifting diets can build support for targets and interventions needed to achieve them (Box 2). Targets can take several forms, including reducing consumption of resource-intensive foods and increasing consumption of healthier and more sustainable foods. While many countries have national nutritional targets and dietary guidelines, few have targets or guidelines that combine nutritional and environmental objectives.^96^ Sweden, Germany, the Netherlands, Brazil, and China are exceptions. Governments can conduct assessments of dietary patterns and trends by age, income, ethnic group, urban/rural, and so on, and benchmark the results with peers. The resulting information, along with WHO guidelines for a healthy diet, can inform national targets, help identify shifts with the greatest potential to generate health and environmental benefits, and establish baselines to monitor progress.

Third, governments can experiment with a range of interventions to shift diets (Table 1). Interventions should target populations that overconsume or under-consume relative to dietary recommendations. To date, governments have typically relied on interventions toward the right side of Table 1, particularly information and persuasion. This is changing. The City of London, for example, has banned new fast-food establishments from opening within 400 meters of schools to help reduce high levels of childhood obesity because 40% of children in London are overweight or obese when they finish primary school.^97^ Government fiscal measures, such as farm subsidies, often support production of resource-intensive foods, such as animal foods and feed. This is also starting to change in some countries, creating opportunities to align with promoting healthier and more sustainable diets. Organisation for Economic Cooperation and Development countries, for example, have halved agricultural subsidies since the 1980s, shifting them from production to the support of environmental or social objectives. Diet-related policies focused on reducing meat and dairy consumption and reducing calorie intake have been tried in several countries, including a “junk-food” tax in Hungary and Mexico, and taxes on sugary drinks in Chile, Mexico, and several US states. Two years after taxes were introduced in Mexico, purchases of sugary drinks fell by 8.2%, while purchases of junk food fell by 6.0%.^98,99^ In Hungary, 40% of junk food manufacturers eliminated or reduced unhealthy ingredients from their products in response to taxes.

**Table 1.** Types of Interventions for Shifting Food Choices.^a^

Type of interventionn	Restricting/promoting access	Fiscal measures	Trade rules	Public procurement	Persuasion/campaigns	Information	Research and development
Examples	Advertising limits Ban on saturated fats or carbonated drinks in schools Limiting fast food chains near schools Promoting availability of desirable foods in public facilities Standards on default options in food services sector	Tax on fat, sugar, meat Subsidies on sustainable foods/vouchers for people on income support Carbon tax Aligning production subsidies with sustainable food goals	Food import/export controls Alignment of domestic support/investment liberalization	Procurement standards and criteria, that is, in hospitals, schools, prisons, and other public facilities	Meatless Mondays	National Dietary Guidelines Food labels, certification programs Education program, that is, in schools, clinics	Funding for research on how to shift diets in different contexts and geographies Technology support for plant-based meat substitutes
Comments	This involves restricting access to unsustainable foods while promoting access to sustainable foods Children and youth are an important target. They are especially influenced by advertising and their habits are still being formed.	Empirical evidence is limited on the effectiveness of fiscal instruments. Taxes can be regressive Combining taxes on less sustainable food with subsides on more sustainable alternatives can be more effective	A convergence toward Western-style diets is linked to industrialized agriculture, global food chains, trade liberalization, and mass media Governments can review their trade rules for alignment with supporting sustainable food choices	Procurement policies can specify food types as well as production methods and location	Public spending on information is likely to be dwarfed by countervailing private sector advertising	Consumers can find package labels difficult to comprehend Product labeling can drive food companies to reformulate products Food choices are largely driven by habit not information	Public funding for research on actions to shift diets tends to be limited even though such actions can deliver multiple benefits to the UN Sustainable Development Goals

^a^ Adapted from Table 6 from the study by Ranganathan et al.^100^

Fourth, governments can ensure coherence among agriculture, health, water, and environmental policies. This is especially important given the potential for synergies between sustainable diets and achievement of other national policy goals, such as health, food security, water, biodiversity, and climate change. One way to facilitate policy coherence is to establish an interdisciplinary cross-agency task force to identify policies and regulations that influence food choices; assess whether they are aligned with promoting sustainable healthy diets; and recommend changes to ensure alignment. Key agencies to involve include agriculture, health, environment, education, forests, water, and the lead agency for implementing the s SDGs. This taskforce can also be charged with setting targets, monitoring progress, evaluating the effectiveness of interventions, and scaling up those that prove effective.

### What Actions Can Business, Especially Food Service Providers, Take?

First, like governments, business can set measurable targets, such as increasing the share of plant source foods in sales relative to animal-source foods or reducing diet-related environmental impacts. The food industry, especially the retail and food services sector, can play an active role in the transition to healthier and more sustainable diets. In addition to supporting environmental goals, shifts to sustainable diets can contribute to commitments around employee or customer health and wellness.

Second, business can experiment with approaches to shifting consumer choices. Shifting behavior requires strategies that work in combination with how and why consumers purchase food. Global food companies are experienced in using behavioral economics and commercial marketing strategies to influence consumer choices. These same strategies can be deployed to promote more sustainable food choices, especially if they improve (or do not negatively impact) profitability. In the food services sector, increasing the share of plant-based proteins can reduce costs, as animal-based ingredients can be more expensive than plant-based foods.^101^

Third, businesses can collaborate with government and civil society to drive shifts to more sustainable diets. The Cool Food Pledge platform convened by World Resources Institute, for example, brings together companies, universities, hospitals, and other public facilities to reduce diet-related GHG emissions by 25% by 2030. If business finds that there is not a financial case for acting, it can call upon governments to enact legislation such that their incentives are aligned with promoting sustainable food choices.

**Figure 5.**
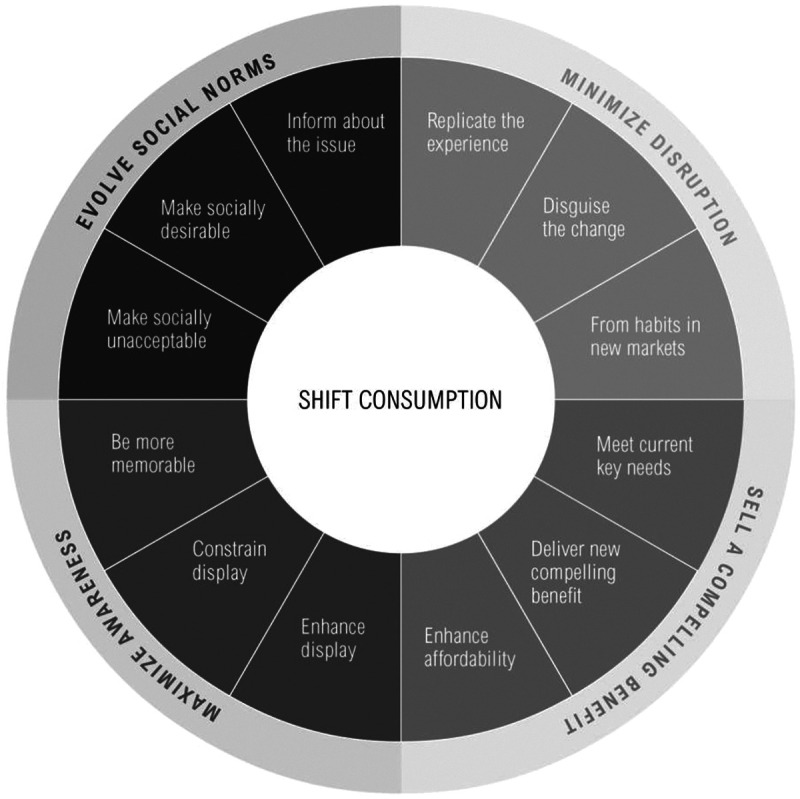
The Shift Wheel provides a framework of 4 complimentary approaches businesses can use to shift consumers to more sustainable diets. Reproduced from Ranganathan et al.^100^ See Figure S2 for a color version available in the online supplement.

The Shift Wheel (Figure 5, S2) provides a framework that businesses can use to shift consumers to more sustainable diets.^100^ It is informed by previous successful shifts and 4 complementary approaches to shift consumption:

- *Minimize disruption* to consumer’s existing habits, including minimizing changes to taste, look, texture, smell, packaging, and the food’s location in a retail environment. For example, companies have created substitutes for animal-based foods from plant- or fungal-based proteins, replicating the taste and texture of chicken, eggs, ground beef, and fish as closely as possible. Others have blended in new ingredients within current formats. In Mauritius, the country’s subsidized cooking oil (ration oil) was reformulated from palm to soybean oil to improve health outcomes and resulted in significant reductions in both saturated fat consumption and blood serum cholesterol levels.^102^ Another way to minimize disruption is to replicate familiar packaging and in-store location. Several soya milk brands, for example, have packaging that looks similar to packaging for fresh milk and have placed their product in retailers’ chillers alongside fresh milk.
- *Sell a compelling benefit* to appeal to consumers, such as health or affordability. For example, Birds Eye marketed its pollock-based fish fingers (fish sticks), a more sustainable alternative to cod fish fingers, as healthier “Omega 3 Fish Fingers.” Language and framing of plant-based foods can encourage meat eaters to choose plant-based foods from a menu. Indeed, changing the names of vegetable dishes to sound more indulgent, for example, “slow-roasted caramelized zucchini bites” increased consumption by 25% to 41% percent compared to traditional names, for example, “zucchini” or healthy names, for example, “nutritious green zucchini.”^103^ Location on a menu also affects choices: putting plant-based foods in a vegetarian box on the menu reduced orders by 56%.^104^
- *Maximize awareness*, including increasing the visibility of a product through memorable advertising or by enhancing its availability and display. Creating memorable advertising involves building consumers’ memory associations with a specific food to increase the probability of it being remembered and purchased. Coca-Cola, for example, is associated with red, its distinctive bottle shape, its logo script, and its ability to refresh on a hot day. Memorable marketing programs for plant-based foods could play an important role in shifting consumption.
- *Evolve social norms*, including adapting or changing the underlying social and cultural norms through informing and educating consumers, along with efforts to make the more sustainable food more socially desirable or the less sustainable food less socially desirable. For some men, eating meat can be a way that they affirm their masculinity.^105^ Consumer studies in Australia have shown that men are more likely to believe that primarily plant-based diets are neither nutritious nor tasty and that these foods do not provide enough energy or protein.^106^ By better barriers to increased consumption of plant-based foods, food companies can design marketing campaigns that shift social perceptions.

## Conclusion

The global food system threatens achievement of international health and environmental sustainability targets. Achievement of these targets will be further threatened by growing populations demanding larger quantities of less healthy and higher impact foods. Finding solutions to this diet, health, and environment trilemma is a global challenge. This challenge needs to be addressed at local to global scales. For example, identifying where, when, and in which countries crops might be most environmentally sustainably grown could contribute to the concept of global seasonality.^107^

Dietary change presents a unique opportunity to meet multiple SDGs. Adopting sustainable healthy diets rich in plant-based foods could slow, and potentially reverse, the growing impact diets are having on human health and the environment, although it must not be assumed that healthy diets will have a low environmental impact.^23,12^ Dietary guidelines must integrate health and environment for sustainable healthy diets to minimize the chance of unintended consequences.^33^ Other changes to the food system, such as reducing food loss and waste, technological adaptation, or changes in food formulation, processing, and preparation, could also improve the environmental sustainability of food systems.^23^ It is, however, unclear which food system changes might be most feasible because economic costs and barriers to implementation to food system changes will vary by context both between and within countries. Such changes should be paired with monitoring efforts to ensure that they improve sustainability without creating unwanted trade-offs.

No single “silver bullet” solution exists to the ongoing and increasing diet-related health environmental sustainability challenges. A combination of coordinated and multisectoral actions will be needed by the public, private sectors, and governments to shift diets, with context specific solutions required to account for the unique challenges and food cultures, dietary preferences, and institutional structures present in each country.

To transition to more environmentally sustainable diets, governments can start by conducting assessments of current dietary patterns and trends by age, income, ethnic group, urban/rural, and so on, and benchmark the results with peers. The resulting information can inform context-specific targets, identify shifts with the greatest potential to generate health and environmental benefits, and establish baselines to monitor progress. Efforts will need to be coordinated across multiple agencies (eg, agriculture, health, education, and environment), given the potential for synergies between environmentally sustainable diets and the achievement of other national policy goals. But if successful, shifting to sustainable healthy diets offers a rare win-win approach for health and environment.

## Boxes

Box 1: Common Food Myths That are not Always True.

a) *How and where a food is produced matters more than the type of food.* Food production matters, but food type is typically a larger indicator of its environmental impact than is how or where it is produced.^20^ The GHG impacts of food processing and transportation are small compared to the emissions resulting from food production. Similarly, the difference in impact of organic and nonorganic foods are often small and vary across environmental indicators (eg, GHG emissions and land use).^21^
b) *Shifting to more plant-rich diets risks people not getting enough protein.* The majority of people are consuming more protein than required to meet their nutritional needs. Consuming more protein is thus not necessarily better, unless an individual is malnourished. International nutritional guidelines recommend adults consume 0.83 g of protein per kg of body weight, or ∼58 g of protein per day for 70 kg adult.^38^ In 2009, the average person in over 90% of the world’s countries consumed more protein than what was recommended in nutritional guidelines, with protein intake in many middle- and high-income nations far exceeding recommendations.^100^ For instance, the average American adult consumed 66% more protein per day in 2012 than the average estimated daily requirement, but 21% of adults still considered themselves protein deficient in a 2014 survey.^108,109^
c) *There is no need to reduce meat consumption, we can just produce it more efficiently.* We need to do both. Increasing efficiency is an essential element of sustainable food systems, but new technology and production methods will likely not be adequate to meet environmental targets.^11^ Given projected growth in meat demand, reducing meat consumption among high-meat consumers is needed for health and environment, and to create space for under-consumers to eat more.

Box 2. Unanswered Questions Related to the Economic Impacts of Dietary Changes.

a) *What are the economic costs and benefits of dietary changes?* The economic benefits of dietary changes are potentially large. Poor dietary quality is the leading health care cost in many middle- and high-income nations. In the United States, for instance, ∼32% of medical expenditures in 2008 resulted from overweight and obesity, cardiovascular disease, or type 2 diabetes.^110^ Shifting to healthier dietary patterns is likely to reduce disease incidence, associated health care costs, and economic losses resulting from poor health.^22^
b) *What is the role of aquaculture in future food systems?* Most of the world’s fisheries are either maximally or overexploited, meaning that there is minimal capacity to sustainably increase fish production from wild fisheries.^111^ Sustainably meeting increased fish demand will need to be met through aquaculture. Aquaculture systems vary widely, potentially having low impacts (eg, unfed filter feeders like mussels) to high impacts (eg, coastal shrimp systems often expand at into mangroves).^19,20^ Finding sustainable aquaculture systems suited for local environmental, economic, and social conditions will be key to meeting growing demand for fish.
c) *What is the health and economic costs of agricultural production?* Most focus on the health and economic impact of agriculture and of food systems is on direct impact of the foods we eat. However, how we produce foods also has health and economic costs. For health, air pollution is the largest environmental risk factor for mortality and poor health, and agricultural activities accounts for ∼20% of premature deaths from air pollution globally.^112^ Similarly, agricultural production has environmental impacts, such as water pollution and biodiversity losses, which reduce the capacity of Earth’s ecosystems to produce food in the future. The health and economic costs of agricultural production are not yet well quantified and warrant exploration. New business models can explore the potential for sustainable food system to contribute to economic stability.

## Supplemental Material

supplemental_figures - The Role of Healthy Diets in Environmentally Sustainable Food Systems supplemental_figures for The Role of Healthy Diets in Environmentally Sustainable Food Systems by Michael Clark, Jennie Macdiarmid, Andrew D. Jones, Janet Ranganathan, Mario Herrero and Jessica Fanzo in Food and Nutrition Bulletin
