# Supplementary material for: The Role of Healthy Diets in Environmentally Sustainable Food Systems
Source: Food Nutr Bull. 2020 Dec 24;41(2 Suppl):S31–58. doi: 10.1177/0379572120953734 (PMC11951467; doi:10.1177/0379572120953734)
Supplement: supplemental_figures - The Role of Healthy Diets in Environmentally Sustainable Food Systems [file supplemental_figures.pdf]

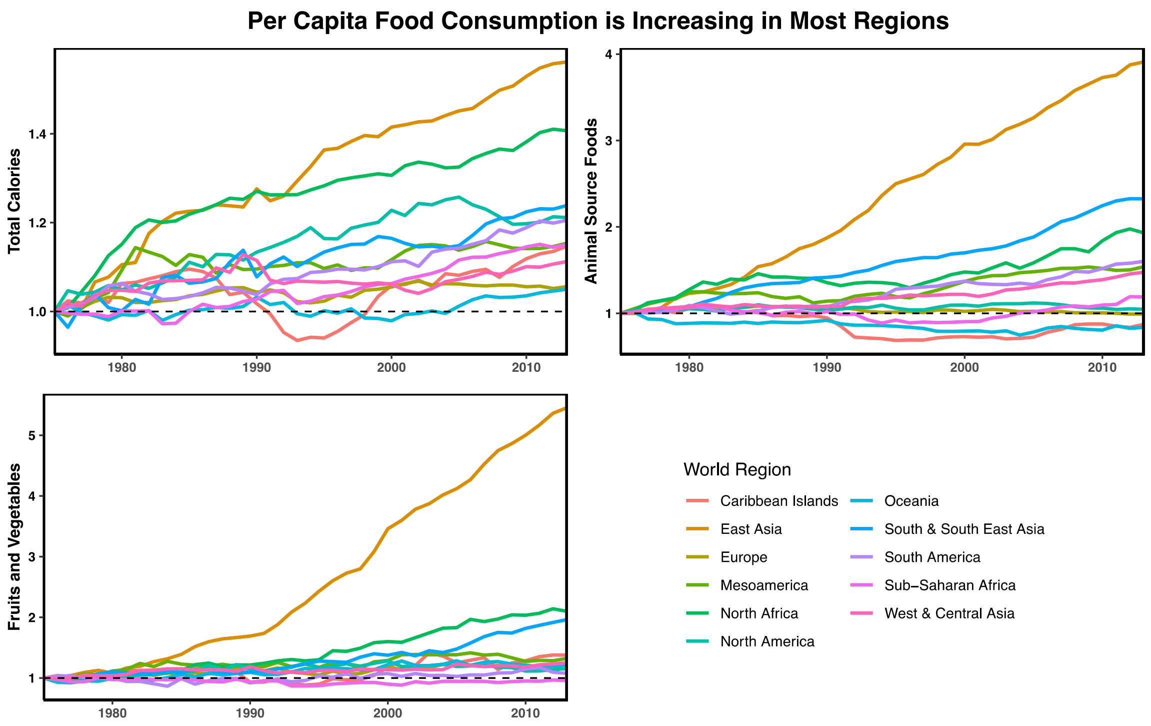

Figure S1. Proportional change in per capita caloric supply, of **(a)** total calories, **(b)** calories from animal-source foods, and **(c)** calories from fruits and vegetables. Lines are colored by world region, with a value >1 indicating an increase in per capita food consumption, whereas a value <1 indicates a decrease in per capita food consumption. Data is from ref [10].

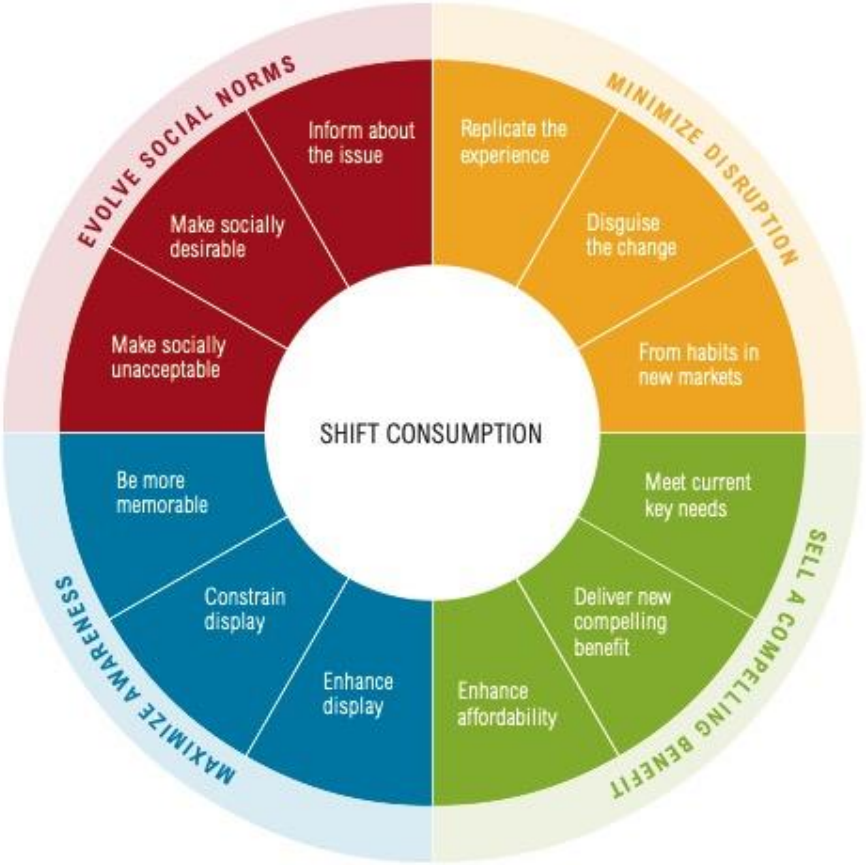

Figure S2. The Shift Wheel provides a framework of four complimentary approaches businesses can use to shift consumers to more sustainable diets. Reproduced from ref [103].
